# The bilocated mind: new perspectives on self-localization and self-identification

**DOI:** 10.3389/fnhum.2013.00071

**Published:** 2013-03-08

**Authors:** Tiziano Furlanetto, Cesare Bertone, Cristina Becchio

**Affiliations:** ^1^Dipartimento di Psicologia, Centro di Scienza Cognitiva, Università di TorinoTorino, Italia; ^2^Centro di Ontologia Teorica e Applicata, Università di TorinoTorino, Italia

**Keywords:** mental bilocation, perspective taking, autoscopic phenomena, virtual reality, minimal phenomenal selfhood

## Abstract

Does the human mind allow for self-locating at more than one place at a time? Evidence from neurology, cognitive neuroscience, and experimental psychology suggests that mental bilocation is a complex, but genuine experience, occurring more frequently than commonly thought. In this article, we distinguish between different components of bilocated self-representation: *self-localization* in two different places at the same time, *self-identification* with another body, reduplication of *first-person perspective*. We argue that different forms of mental bilocation may result from the combination of these components. To illustrate this, we discuss evidence of mental bilocation in pathological conditions such as heautoscopy, during immersion in virtual environments, and in everyday life, during social interaction. Finally, we consider the conditions for mental bilocation and speculate on the possible role of mental bilocation in the context of social interaction, suggesting that self-localization at two places at the same time may prove advantageous for the construction of a shared space.

In daily life the self is typically tied to one place at a given point in time and this place coincides with the body. As Husserl puts it: “I do not have the possibility of distancing myself from my body, nor it from me” (Husserl, 1952/1989). Self-experience, however, is not always constrained by the body: empirical research into self-related disorders and full-body illusions demonstrates that the spatial unity between body and self can be temporarily suspended. For seconds, and more seldom minutes, neurological and psychiatric patients may experience themselves to be localized at, and to see from, a location outside their physical body (Blanke and Mohr, 2005). A similar experience might be experimentally induced in healthy subjects using mirrors or simple virtual reality devices (Lenggenhager et al., 2007, 2009).

Where does the self localize during such experiences? Out-side the bodily borders? At the location of the physical body? Does the human mind allow for locating at more than one place at the same time? In this paper we focus on this latter question, and consider the spatial and temporal dynamics of the self-localization process. In particular, we discuss the possibility that the self might be distributed over two spatially distinct places at the same time.

Based on the concept of “minimal phenomenal selfhood” (MPS; Blanke and Metzinger, 2009), our contention is that *mental bilocation*, i.e., localization of the self at two distinct places at the same time, is not a single perceptual experience but can be broken down into different components: *self-localization* in two different places at the same time, *self-identification* with another body, and reduplication of *first-person perspective*. Different forms of *mental bilocation* may result from the combination of these components. In this article we will discuss three instances of mental bilocation in which the above mentioned components appear differentially present: *heautoscopy*, *virtual presence*, and *perspective taking* (see Table 1). Although mental bilocation in its complete form is only experienced during heautoscopy, incomplete forms of mental bilocation may be experienced during immersion in virtual reality, and in everyday life, during spatial perspective taking.

**Table 1:** **Instances of mental bilocation in which the three MPS components are differentially present**.

	**Self-localization in two different places at the same time**	**Self-identification with another body**	**Reduplicated first-person perspective**
He-autoscopy	√	√	√
Virtual presence	√	√	
Spatial perspective taking	√		

Incidences of bilocation are reported in many different cultures at many times. We propose that these reports are rooted in the complex experience of being mentally at two places at the same time, an experience which—we argue—is more frequent than commonly thought and might play an important role in the construction of a shared space.

## Heautoscopy: the bilocated self

Heautoscopy, i.e., the encounter with one's double (Menninger-Lerchenthal, 1935), is a rare multimodal illusory experience characterized by the reduplication of one's own body and self (Blanke and Mohr, 2005). As in other forms of autoscopic phenomena such as autoscopic hallucination, during heautoscopy the patient sees a double of herself in the extrapersonal space. This double, however, is not a mere image or visual hallucination. The self can be experienced as being at the position of physical body (body-centered frame of reference) and, simultaneously or in rapid alternation, at the position of a reduplicate body in the extrapersonal space (alter-body-centered frame of reference; Lopez and Blanke, 2007). Self-location and first-person visual-perspective may alternate between an embodied and a disembodied location and it might be difficult for the subject to decide where the self is localized (Brugger, 2002). As illustrated by the following example, the patient may indeed experience to be at both positions at the same time:

The patient has the immediate impression as if she were seeing herself from behind herself. She felt as if she were ‘standing at the foot of my bed and looking down at myself’. Yet, […] the patient also has the impression to ‘see’ from her physical visuo-spatial perspective […]. Asked at which of these two positions she thinks herself to be, she answered that ‘I am at both positions at the same time’ (Patient 2b, Blanke et al., 2004).

Heautoscopic experiences are often associated with changes in the awareness of one's body. Patients may, for example, report abnormal vestibular sensations such as abnormal lightness or hollowness of the body which may feel “just like an empty shell after the chick has hatched” (Lukianowitz, 1958). With increasing body depersonalization, there is an increase in personalization of the illusory double, to the point that the patient may wonder whether it is the physical body or rather the reduplicated body which contains the real self (Brugger, 2002). Not only self-localization and first-person perspective, but also self-identification may therefore be experienced as “split in two parts” (Brugger et al., 1994).

**Figure 1:**
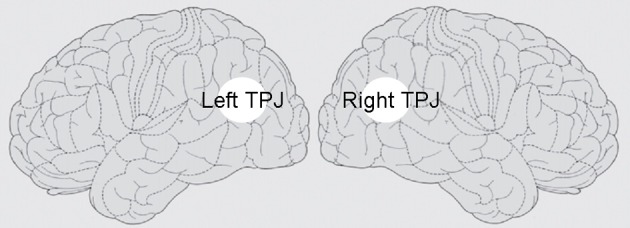
**Hypothesized neural correlates of mental bilocation.** A plausible candidate for the neural substrate of mental bilocation is the point of convergence of the angular, superior temporal, and upper marginal gyri at the temporo-parietal junction (TPJ). Brain damage or brain dysfunction is localized at the TPJ in neurological patients experiencing out-of-body and he-autoscopic phenomena (Blanke et al., 2004). In healthy subjects, interference with the TPJ by transcranial magnetic stimulation impairs mental own body transformation (Blanke et al., 2005). Neuroimaging studies support the role of the TPJ in vestibular processing, integration of multisensory body related information (Leube et al., 2003), mental imagery using disembodied self location (Arzy et al., 2006), and experience of presence in virtual environments (Jäncke et al., 2009; Ganesh et al., 2012). Finally, cortex at the TPJ has also been involved in visuo-spatial perspective taking. For instance, TPJ is the classical lesion site in patients with egocentric spatial neglect, a clinical condition which has been shown to disturb the patient's egocentric representation of space (Halligan et al., 2003). Neuroimaging studies in healthy subjects have revealed TPJ activation during visuo-spatial perspective changes (Zacks et al., 2003; Aichhorn et al., 2006; David et al., 2008).

Heautoscopy of neurological origin has been related to pathological activity patterns primarily localized at the temporo-parietal-junction (TPJ; Blanke and Mohr, 2005; see Figure 1). In healthy subjects, a similar duplication of the self with two distinct and active roles can be experienced during REM sleep, when the dreamer has both the role of the chief character and that of the observer or plays different protagonist roles (the pursuer and the pursued person; Cicogna and Bosinelli, 2001). As heautoscopy, these situations may involve oscillations of first-person perspective and uncertainty relative to perception, feelings, and emotions (Occhionero and Cicogna, 2011). Because REM sleep involves considerable deafferentation and reduction of incoming peripheral vestibular information, this supports the implication of the vestibular cortex situated at the TPJ in the generation of heautoscopic experiences.

## Virtual presence: bilocation in virtual reality

A common metric of the quality of virtual environments is the degree to which the virtual environment creates in the user the illusion of presence—the subjective experience of being in one place when physically situated in another (Heeter, 1992; Witmer and Singer, 1998). Similar feelings of presence at distant places may arise during teleoperations (Ruff et al., 2002), in telepresence videoconferencing (Anderson et al., 2001), and during immersion in cybertherapy settings (Price and Anderson, 2007).

Telepresence and virtual presence are generally thought to imply a “departure” from the physical environment and an “arrival” in the mediated environment (Sadowsky and Stanley, 2002). Little, however is, known about the temporal and spatial dynamics of these self-localization processes. May individuals perceive themselves localized “here” and “there” at the same time? To investigate variations of presence over time, Wissmath et al. (2011) employed a two-dimensional continuous measurement paradigm based on handheld slider. Participants were exposed to a virtual rollercoaster simulation. In two separate rides participants were asked to use the slider to report second by second on a scale from 0 to 100% to what extent they felt located in the immediate physical environment and to what extent they felt located in the mediated virtual environment. The results revealed that participants were able to integrate both localizations and distributed their presence in both realities: 30 s after the onset of the presentation they localized themselves in both the immediate and the mediated environment. Most noteworthy, the findings indicated an almost perfect inverse relationship between self-localization in the two environments over time: the stronger self-localization in the mediated environment at a certain point in time (e.g., 60% after 60 s), the weaker the feeling of being localized in the immediate environment at the corresponding time during the other ride (40% after 60 s).

A similar pattern of bilocated self-localization, may be experience in collaborative virtual environments, such as online role playing games, in which users are represented by a virtual alter-ego, commonly referred to as an avatar. A unique feature of online role playing games is that players can navigate the game world and control their avatar from a first- or third-person perspective. In the third-person perspective, players continuously have a visual percept of their avatar while they control its movements. This gaming mode may offer a non-pathological form of self-experience in which, similarly to heautoscopy, gamers identify with their avatar from a third-person perspective (Ganesh et al., 2012). Sense of agency and control over the avatar facilitate this kind of self-identification (Pearce and Artemesia, 2006; Weibel et al., 2008) and some gamers seem indeed to identify more strongly with their avatar than with their real self (Bessiere et al., 2007; Yee et al., 2009). In heautoscopy, self-localization can be either centered in the physical body or in the illusory body or in both. This may also be the case in third-person perspective gaming: self-location may alternate back and forth between the player's body and the avatar body, or, may be present in both the gamer and the avatar simultaneously. Notably, compared to self-referencing, avatar-referencing during online role playing games has been shown to generate more activity in the angular gyrus at the TPJ (Ganesh et al., 2012; Figure 1). Activity in this area is significantly correlated with the duration of daily online gaming and has been shown to be stronger in internet addicted adolescents compared to non-addicted adolescents (Kim et al., 2012).

Further support for the hypothesis that mental bilocation may be experienced in virtual environments is provided by full-body illusions research, in which the unity of self and body is experimentally manipulated by exposing participants to conflicting multisensory cues by means of mirrors or simple virtual reality devices (Ehrsson, 2007; Lenggenhager et al., 2007, 2009; Petkova and Ehrsson, 2008; Petkova et al., 2011; see also, Connors et al., 2012 for the use of hypnosis to recreate mirrored-self misidentification delusion). In Lenggenhager et al. (2007) for example, participants viewed the back of their body filmed from a distance of 2 m and projected onto on a 3D head-mounted video. The participants' back was stroked during 1 min either synchronously or asynchronously with respect to the virtually seen body. Self-localization was measured by passively displacing the blindfolded participants immediately after the stroking and asking them to return to their initial position. In the synchronous stroking condition, participants systematically mislocalized themselves toward the virtual body (Lenggenhager et al., 2007, 2009). This drift toward the virtual body might arise from the simultaneous encoding of self-location with respect two competing frames of reference: a framed of reference centered at the location of the physical body, and a frame of reference centered at the location of the virtual body. Being constrained to indicate one single localization of the self, participants might localize themselves at a “compromise” location between the two competing location (Wissmath et al., 2011).

## Spatial perspective taking: social bilocation

Converging evidence from the field of social neuroscience suggests that people relate knowledge of their own body to understand other people's behavior (Grafton, 2009). Accordingly, understanding others' actions, intentions, and emotions have been proposed to rely on mechanism of embodied simulation (e.g., Becchio et al., 2012). Using a spontaneous motor paradigm, Thirioux et al. (2010) investigated whether individuals also embody others' localizations, mentally locating themselves in the position of other bodies during social interaction. Participants observed a life-sized virtual tightrope walker leaning to her left or right on a rope. In a first experiment task, they interacted spontaneously with the tightrope walker by leaning when she was leaning. In a second and third experiment task, they were instructed to lean when the tightrope walker was leaning by either imagining their body in the position of the tightrope walker' body (rotation) or imagining their body at their actual body position (mirror reflection). Interaction tilt patterns were indistinguishable from rotation tilt patterns at both the motor and the neural level, suggesting that during interaction participants spontaneously located themselves in the walker's body position.

Further evidence that social situations may influence self-localization comes from studies investigating spatial perspective taking, i.e., the ability to adopt the spatial perspective of another person (Mainwaring et al., 2003; Zacks et al., 2003; Aichhorn et al., 2006; David et al., 2008; Frischen et al., 2009; Tversky and Hard, 2009; Zwickel, 2009; Schober and Carstensen, 2010; Zwickel and Müller, 2010; Shelton et al., 2012). In an egocentric frame of reference, objects and locations are encoded with respect to one's own body. Tversky and Hard (2009) have shown that the mere presence of another person in the position to act on objects induces a good proportion of respondents to describe spatial relations from the other person's point of view. This effect is not limited to type of descriptors used, but, as confirmed by recent neuropsychological evidence, entails a spatial remapping of objects and locations with reference to the other person's body (Becchio et al., 2013). Patients affected by left egocentric neglect—a failure in attending and reporting stimuli on the left side of the body-centered space—were asked to describe different arrays of objects from their own perspective, from the opposite perspective, or from the point of view of another person actually seated in front of them. Items presented on the left side and omitted when report was required from the first-person perspective could be recovered when patients assumed a different spatial perspective. Critically, no left neglect was observed when report was required from the perspective of another person actually present in the scene, suggesting that objects and locations were remapped within an alter-centric frame of reference.

These findings suggest that in social situations people may overcome their own position in space to localize themselves at the position of the other person. But do people actually disengage from an egocentric frame of reference when they represent the scene from the perspective of another person? Could social situations involve the simultaneous activation of multiple perspectives or reference frames? Samson et al. (2010) report that observers are slower to make self-perspective judgments when the scene includes an avatar looking from a different perspective. In a series of experiments participants saw a picture of a room with a human avatar facing one of the walls, and with red discs displayed on the walls. In the consistent perspective condition, both the participant and the avatar could see the same amount of discs. In the inconsistent perspective condition, the participant and the avatar each saw a different amount of discs. Participants were then asked to judge how many discs could be seen, either from their own perspective or from the avatar's perspective, while ignoring the irrelevant perspective. Slower responses and more errors in the inconsistent perspective condition compared to the consistent perspective condition were found both when participants judged the avatar's perspective and when they judge their own perspective. This indicates that, just as they were influenced by their own visual perspective when judging what the avatar saw (egocentric intrusions), so they could not prevent themselves from processing the avatar perspective when judging their own visual experience (alter-centric intrusions). Both perspectives appeared therefore to be processed at the same time (Samson et al., 2010). Surtees et al. (2012) and Surtees and Apperly (2012) report a similar effect of simultaneous activation of multiple frames of reference in children as young as 6 years old. When evaluating the appropriateness of statements describing the position of two objects (a ball and a reference object that was either a doll or a model chair), children 7–11 years old showed sensitivity to both an egocentric frame of reference and an object intrinsic frame of reference. As for adults, anchoring to the reference object was stronger when the reference object was a social object (doll).

## Can we mentally bilocate?

The aforementioned evidence suggests that mental bilocation is a complex but genuine experience (see Table 1). In heautoscopy, self-location and first-person perspective are at the position of the physical body and, simultaneously or in rapid alternation, at the position of the heautoscopic body. Moreover, self-identification can either refer to the physical body, to the autoscopic body, or both. All three-dimensions of minimal selfhood—self-localization, self-identification, and first-person perspective—are therefore bilocated (Blanke and Metzinger, 2009).

Taking the perspective of another person, in contrast, seems to involve bilocated self-localization but not dissociation in two objects of identification: people locate themselves at the position of the other person, but do not identify with the other person's body. Furthermore, although spatial positions might be encoded within an alter-body centered reference frame, at the phenomenal level, the world is still perceived from a unitary first-perspective originating with the physical body. In virtual reality, subjects may experience different forms of mental bilocation. Depending on the characteristics of the mediated environment, mental bilocation may vary along MPS dimensions, from distributed self-location over two places to transient identification with virtual alter-ego. Factors that may influence mental bilocation in virtual settings include: the variety of sensory stimulation achievable, the pictorial realism, the possibility to act in real time upon the virtual environment, the representation of participant's body in the virtual space, the presence of others, the visual perspective (Coelho et al., 2006). A recent study by Petkova et al. (2011), suggests that this latter factor—visual perspective—together with the presence of a sufficiently humanoid body, may be critical to trigger self-identification with the artificial body in full-body illusions experiments. Participants self-identify with the artificial body when the artificial body is seen from a first person-perspective, i.e., as though directly looking down at one's body. Self-identification is absent or significantly weaker (in terms of both subjective reports and physiological response to a threat applied to the artificial body abdomen), when the artificial body is seen from a third person-perspective. Independently from visual perspective, no illusory body swapping is observed when the artificial body is substituted with a rectangular object, suggesting that only objects that look like a human body can be “owned” (Petkova and Ehrsson, 2008).

## Why bilocate?

The deep meaning of embodied cognition has been proposed to lie in disembodied thought (Tversky, 2005; Tversky and Hard, 2009). Mental bilocation enriches this view suggesting that not only the self can be located outside the physical body: it can be located at two places at the same time. But why do we mentally bilocate?

There are at least two ways to make sense of this “why-question.” One way is to ask what are the necessary and sufficient neurofunctional conditions for mental bilocation to take place (Metzinger, 2009; see Figure 1). In this connection, pathologically and experimentally induced states of bilocation are a particular relevant target of investigation as they provide insights into the brain mechanism for altered self-localization, self-identification, and first-person perspective taking. Mental bilocation, however, is not only observed under pathological or artificial conditions: it is experienced, in the form of bilocated self-localization, in everyday life during social interaction. This raises a second why-question: what is mental bilocation for? Why do we spontaneously bi-localize when we interact with others?

In these task described earlier (e.g., Samson et al., 2010), bi-localization made individual performance worse. Adopting a bilocated self-localization may, however, prove advantageous during online social interaction (Frith, 2012). Understanding others' actions, anticipating what they will do, and, at the same time, planning an appropriate action in response require to localize objects both with respect one's body and with respect to another person's body. By changing the way spatial events are encoded, mental bilocation may play an important role in construction of a shared interpersonal space. Through mental bilocation, people may escape the imposed body-centeredness and “invariable perspective” (Merleau-Ponty, 1945) of their physical body and circumvent the computational difficulties inherent in any interpersonal mapping (Bråten and Gallese, 2004). On this view, mental bilocation may be more than an “alteration” of normal selfhood (Blanke and Metzinger, 2009): it may instantiate an important interpersonal function, enabling the simultaneous activation of multiple frames of reference, before using inhibition to select the most appropriate perspective (Gallese, 2009; Surtees et al., 2012).

There is now considerable evidence that suggests that there is an implicit form of mentalizing through which we can take account of the mental states of others during joint action (Frith and Frith, 2006; Sebanz and Knoblich, 2009). The knowledge and desires of others are not explicitly represented as mental states. Rather, the mental states of others are taken into account automatically by altering the saliency and representation of objects and actions that are at the focus of joint attention (Frith, 2012). Mental bilocation may contribute to this process favoring the on-line integration of self- and other-centered representations. The “selfhood” we readily attribute to others, the inner feeling of “being-like-you” triggered by our encounter with others may themselves depend on the possibility of being at two places—at the position one's physical body and at the position of the other person's body—at the same time.

### Conflict of interest statement

The authors declare that the research was conducted in the absence of any commercial or financial relationships that could be construed as a potential conflict of interest.
